# Supplementary material for: Forecasting COVID-19 Severity by Intelligent Optical Fingerprinting of Blood Samples
Source: Diagnostics (Basel). 2021 Jul 21;11(8):1309. doi: 10.3390/diagnostics11081309 (PMC8392709; doi:10.3390/diagnostics11081309)
Supplement: Supplementary file 1 [file diagnostics-11-01309-s001.zip › diagnostics-1291233-supplementary.pdf]

Supplementary Information

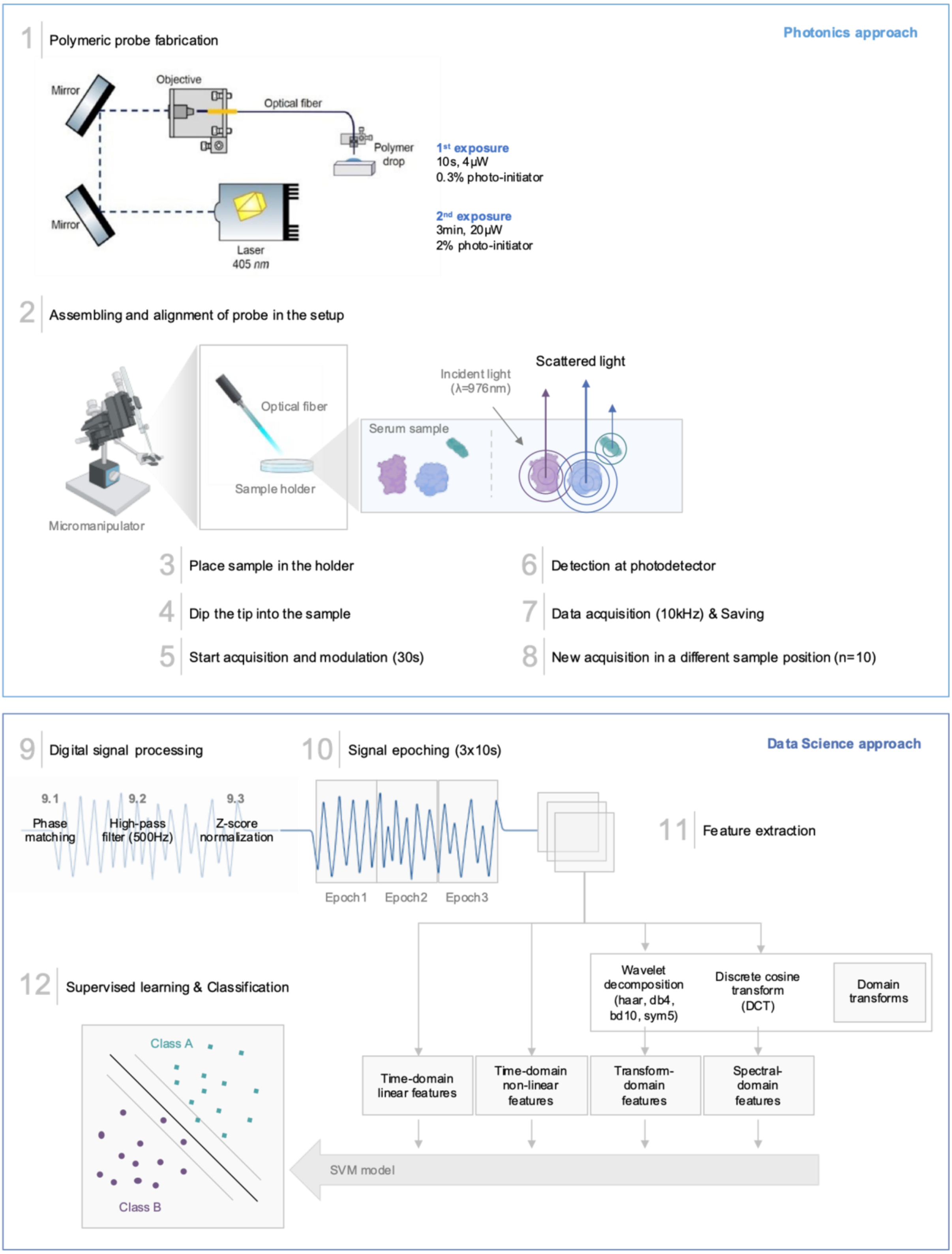

Figure S1. Integrated perspective of the photonics and data science methodology applied in this study.

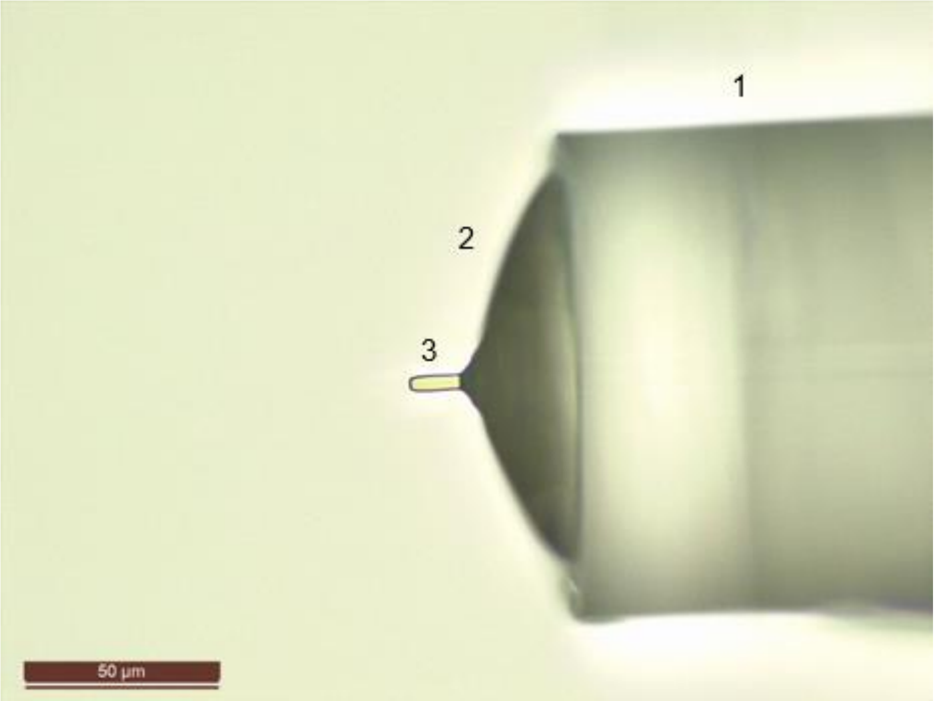

Figure S2. Brightfield image of the probes used to acquire the backscattered signal. Probes are polymeric structures built on the apex of a cleaved optical fiber (1); a dome-like structure (2) was used to protect the in-house produced polymeric lens (3).

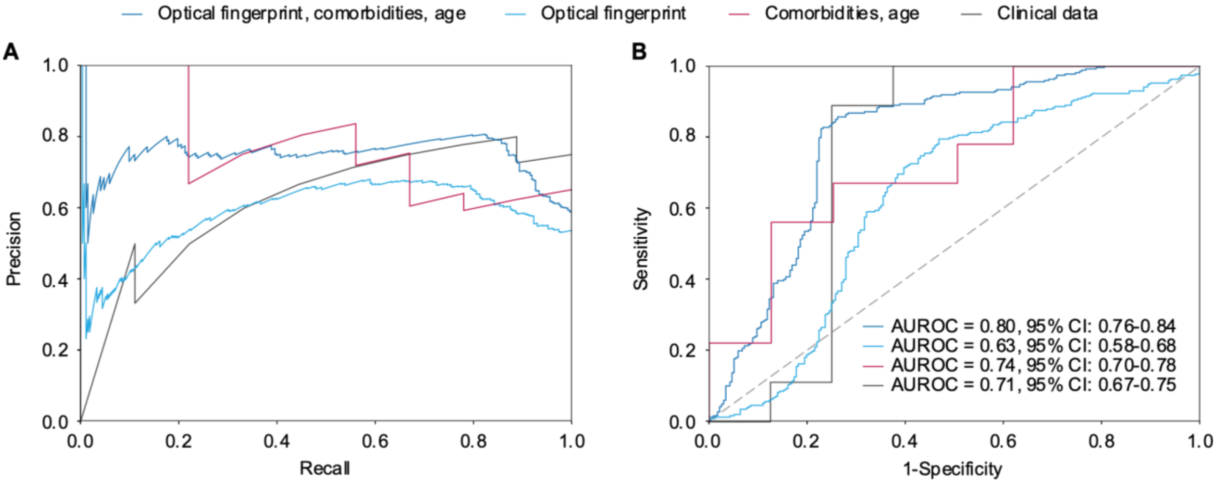

Figure S3. Comparative performance of the machine learning severity prediction models. (A) PR plot showing the positive predictive value (precision) against the sensitivity (recall) for each model. **(B)** ROC curve showing the trade-off between sensitivity and specificity using each model. The diagonal dashed line represents a model with no discrimination. The AUROC with its 95% confidence interval is shown in the plot. Both PR and ROC curves were obtained from the test dataset. AUROC: area under the receiver operating characteristic curve; CI: confidence interval.

A

Variable	COVID *(n=19)*	Non-COVID *(n=19)*	*P* value
Age (y), *median (IQR)*	78 (57-83)	42 (28-59)	**0.0030**
Gender (Male)	14 (73.7%)	7 (36.8%)	**0.0489**

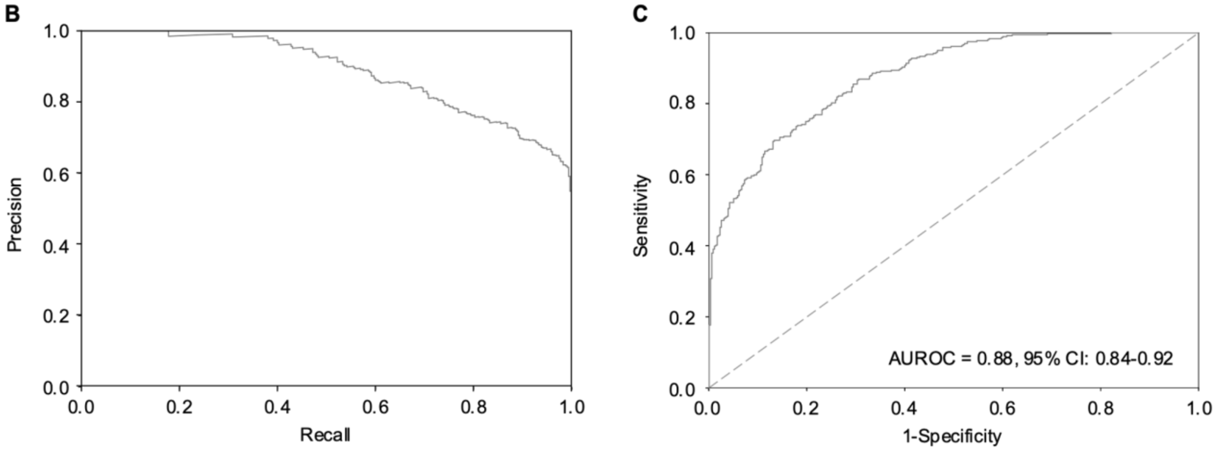

D

Metric	Value (95% CI)
**AUC**	0.88 (0.84-0.92)
**Accuracy**	76.7% (72.0-80.9)
**Sensitivity**	80.0% (73.4-85.6)
**Specificity**	78.6% (66.2-79.6)
**Precision**	75.0% (68.3-81.0)
**NPV**	73.3% (71.6-84.5)

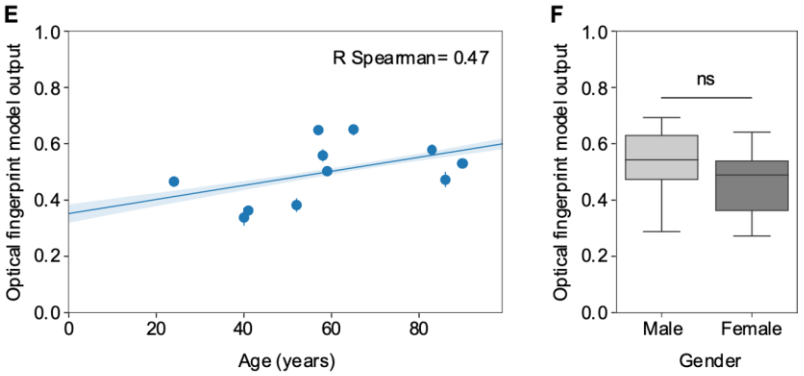

Figure S4. Distinguishing COVID-19 patients from non-COVID-19 patients with similar symptoms using optical fingerprinting. (A) Demographic features of patients included in the COVID-19 and non-COVID-19 groups. **(B)** PR plot showing the positive predictive value (precision) against the sensitivity (recall) of the optical fingerprint model. **(C)** ROC curve showing the trade-off between sensitivity and specificity using the optical fingerprint model. The diagonal dashed line represents a model with no discrimination. The AUROC with its 95% confidence interval is shown in the plot. Both PR and ROC curves were obtained from the test dataset. **(D)** Statistical performance measures of the optical fingerprint model calculated in the testing dataset. Binomial (AUROC) or Clopper-Pearson (accuracy, sensitivity, specificity, and precision) confidence intervals were calculated in the testing dataset. **(E)** Correlation by linear regression between the optical fingerprinting numeric output and patient age. Spearman correlation coefficient was calculated. **(F)** Association between optical fingerprinting numeric output and patient gender. IQR: interquartile range; AUROC: area under the receiver operating characteristic curve; CI: confidence interval; ns: non-significant.

Table S1. Time- and frequency-domain features extracted from the sample backscattered signal to generate the optical fingerprints

Type	Group	Feature
Time domain	Linear	Standard Deviation
		Interquartile range
		Kurtosis
		Skewness
		Mean
		Root mean square
		Signal power
		Entropy
		Root sum of squares level
		Area under the curve histogram
	Non-linear	Approximate entropy
		Singular value decomposition entropy
		Petrosian fractal dimension
		Higuchi fractal dimension
		Detrended fluctuation analysis coefficient
		Hurst Exponent
		Hjorth complexity
		Hjorth mobility
Frequency domain	Transform domain	1st – 30th DCT coefficient
		Number of DCT coefficients that capture 98 % of the original signal
		DCT Entropy
		Total DCT Area Under Curve
		1st – 10th Hilbert peak
		Number of Hilbert coefficients that capture 98 % of the original signal
		Haar Relative Power 1st level – 6th level
		Db10 Relative Power 1st level – 6th level
		Symlet Relative Power 2nd level - 6th level
		Db4 Relative Power 2nd level – 6th level
	Spectral domain	Spectral contrast max
		Spectral roll-off frequency SD
		Spectral roll-off frequency mean
		Spectral roll-off frequency max
		Spectral flatness SD
		Spectral flatness mean
		Spectral flatness max
		Spectral centroid SD
		Spectral centroid mean
		Spectral centroid max

Table S2. Features used in age and comorbidities-based and age, comorbidities and laboratory-based data models

Type	Feature	Description
Demographic	Age	Numeric variable, years
	Gender	Categorical variable (0=male; 1=female)
Clinical	Kidney disease	Numeric variable, count of kidney-related diseases reported in patient clinical records
	Cardiovascular disease	Numeric variable, count of cardiovascular diseases reported in patient clinical records
	Immunosuppression	Numeric variable, count of diseases mentioned in patient clinical records that might induce immunosuppression
	Diabetes	Categorical variable, whether the patient suffers from diabetes (0=no diabetes; 1=diabetes)
	Respiratory disease	Numeric variable, count of respiratory comorbidities reported in patient clinical records
	Obesity	Categorical variable, whether the patient suffers from obesity (0=no obesity; 1=obesity)
	Number of comorbidities per patient	Numeric variable, total number of comorbidities reported in patient clinical records
	C-reactive peptide	Numeric variable, concentration of C-reactive peptide detected in blood
	Leukocyte count	Numeric variable, count of leukocytes detected in blood
	Lymphocyte count	Numeric variable, count of lymphocytes detected in blood
	IgG detection	Categorical variable, IgG antibodies detection (0=absence; 1=present)

Table S3. SVM model parameters tuned during classifier training stage for model optimization

Parameters	Model		
	Optical fingerprinting	Comorbidities, age	Clinical data
Kernel	Linear	Radial Basis Function	Radial Basis Function
Gamma	100.00	0.60	0.01
C	0.22	0.01	0.22

Coefficients	Stacking Model	
Coefficient 0	4.03	
Coefficient 1		6.10

Table S4. Random forest model parameters tuned during classifier training stage for model optimization

Parameters	
Method for sampling data points	Bootstrap
CCP alpha	0
Class weight	Balanced
Criterion	Gini
Maximum number of levels in each decision tree	2
Maximum number of features considered for splitting a node	6
Minimum impurity decrease	0
Minimum number of data points allowed in a leaf node	16
Minimum number of data points placed in a node before the node is split	2
Minimum weight fraction leaf	0
Number of trees in the forest	120

Extended methods

Description of the backscattered signal features

1. **Time-domain features**

Time domain linear features

Time domain metrics such as mean, standard deviation, root mean square, signal power, root sum of squares level (RSSQ), skewness, kurtosis, interquartile range, and entropy were used, given its adequacy in differentiating types of periodic signals. Statistical time-domain parameters have been used to identify tumour cell clusters in cell lines and to identify different objects through the backscattered signal in underwater conditions.[1,2]

For instance, skewness reflects the distribution symmetry degree, while kurtosis quantifies whether the shape of the data distribution matches the Gaussian distribution. Both have been widely used in several signal processing approaches, for quantifying how far, in statistical terms, the evaluated sample distribution is from a normal one.[3] These features have been used as relevant markers for diagnosing mild cognitive impairment (MCI) by performing different mental tasks in patients, using functional near-infrared spectroscopy (fNIRS). The parameter skewness revealed a significance difference for the brain regions analyzed.[4] A similar study involving this technique was made to identify possible biomarkers of pain.[2]

Time domain non-linear features

Non-linear features are useful to describe the complexity and regularity of a signal and are often used to describe the phase behaviour of predominantly stochastic signals, such as EEG.[5] A total of 8 non-linear features were considered: approximate entropy, singular value decomposition (SVD) entropy, Petrosian fractal dimension, Hurst exponent, Detrended fluctuation analysis (DFA), Higuchi fractal dimension, Hjorth complexity and mobility.

- Approximate entropy

Approximate entropy is an indicator of the complexity of the time series, which have been useful to detect several pathological or physiological conditions.[6-9] This technique quantifies the amount of regularity and the unpredictability of fluctuations over time-series data.

- Singular value decomposition entropy

SVD entropy is an indicator of the number of eigenvectors that are needed for an adequate explanation of the data set.[10] In other words, it measures the dimensionality of the data.

A fractal dimension is a ratio providing a statistical index of complexity comparing how detail in a pattern changes with the scale at which it is measured. It has also been characterized as a measure of the space-filling capacity of a pattern that tells how a fractal scales differently from the space it is embedded in; a fractal dimension does not have to be an integer. It is a highly sensitive measure for the detection of hidden information contained in physiological time series, because it performs well on turbulent and irregular time series data and has been widely used to extract quantitative features from biomedical signals, including imaging and EEG.[11,12]

- Petrosian fractal dimension

Petrosian's algorithm provides a fast computation of the fractal dimension of a signal by translating the series into a binary sequence.

- Higuchi fractal dimension

Higuchi is an algorithm for measuring fractal dimension of time series and is used to quantify complexity and self-similarity of signal.[13] Higuchi’s fractal dimension originates from chaos theory and for almost thirty years it has been successfully applied as a complexity measure of artificial, natural, or physiological signals. Higuchi’s method has proven to be a good numerical approach for rapid assessment of signal nonlinearity and it may encompass all information about the dynamic data generation process.

The Higuchi dimension has long been used in applications of clinical neurophysiology to measure the complexity of neuronal activity, or to measure the signal length of various biophysical signals, *e.g.*, EEG and MEG.[14-16]

- Detrended fluctuation analysis coefficient

DFA is a method for quantifying fractal scaling and correlation properties in the signal. The main advantage of this method is that it distinguishes intrinsic fluctuation generated by the system from that caused externally.[13]

- Hurst exponent

The Hurst exponent measures the “long-term memory” of a time series. It can be used to determine whether the time series is more, less, or equally likely to increase if it has increased in previous steps. It originates from H.E. Hursts observations of the problem of long-term storage in water reservoirs.[17]

- Hjorth complexity & Hjorth mobility

Bo Hjorth proposed a mathematical method to describe an EEG trace quantitatively, which has been widely applied to various EEG-based problems.[18,19] The mobility parameter is the square root of the ratio between the variance of the first derivative and the variance of the signal. The complexity parameter represents the changes of the signal frequencies. The Hjorth complexity is the ratio between the Hjorth mobility of the first derivative of the signal and the Hjorth mobility of the signal. This parameter is dimensionless and, due to the non-linear calculation of standard deviation, quantifies any deviation from the sine shape. The value converges to 1 if the signal is more similar.

2. Frequency transform-domain features

Regarding the frequency-domain analysis of the backscattered signal, three sets of features were extracted: Discrete Cosine Transform (DCT) parameters, Wavelet derived coefficients and spectral features.

Discrete Cosine Transform

The DCT, applied to each epoch of the backscattered signal, captures minimal periodicities of the signal, without injecting high-frequency artifacts in the transformed data. Besides being highly adequate to short signals, it is highly attractive for this type of problems which require to differentiate target classes, because DCT coefficients are uncorrelated. Thus, they can be used as suitable features for characterizing each peptide class. Additionally, the DCT is able to embed most of the signal energy into a small number of coefficients. The first *n* coefficients of the DCT of the scattering echo signal are defined by the following equation:[20]

$$E_i^{DCT}[l] = \sum_{k=0}^{N-1} \varepsilon_i[k] \, cos \, cos \left[\frac{\pi l(2k+1)}{2N}\right], for\ l = 1, \dots, n$$

where ε_i is the signal envelope estimated using the Hilbert transform. The following features were extracted from DCT analysis: the number of coefficients needed to represent about 98% of the total energy of the original signal, the first 30 DCT coefficients, the Area Under the Curve (AUC) of the DCT spectrum for all the frequencies before the modulation frequency (1 kHz) and, the entropy of the DCT spectrum. As an example, the DCT feature has been used in scattering data collected from different species of saltwater fish to capture approximately periodic structures in the echo envelope that may result from scattering from internal structures in the fish body.[20]

Hilbert Transform

A similar analysis to the DCT transform was conducted using the Hilbert transform. When applied to the signal, the Hilbert transform produces its analytical real-valued representation. The 10 highest amplitude peaks of the Hilbert transformed signal were used as features, as well as the number of

coefficients needed to represent about 98% of the total energy of the original signal. The first Hilbert coefficient corresponds to the highest peak in the analytic signal and can give important information about the phase of the signal.[21]

Wavelet Transform

Some parameters based on the information extracted from Wavelet analysis of each original signal portion were also considered as features, due to their simplicity and their successful application to decompose backscattered signals in underwater scenarios.[20,22] By applying wavelet packet decomposition, it is possible to extract, in each frequency band, certain tonal information from the original signal depending on the frequency range and content of the backscattered signal.[22] To achieve this, a suitable mother Wavelet is chosen to be used as a prototype to be compared with the original signal and extract frequency subband information.[23] Four mother Wavelets – Haar, Daubechies (Db10 and Db4) and Symlet - were selected to characterize the backscattered signal portions. The Haar wavelet was selected due to its simplicity and computational speed; the Daubechies wavelets display a better approximation of smooth functions;[23] and, the Symlet wavelets have been used to decompose the signal into five time–frequency subbands to recognize epileptic EEG states. This feature can reduce the phase distortion in the analysis.[24]

Frequency spectral-domains features

Spectral features characterize the power spectrum of the signal, *i.e.*, the distribution of power across the frequency components composing that signal. It is obtained using the Fourier Transform. Four measures were derived from the spectrum: spectral flatness, spectral centroid, spectral contrast, and spectral roll-off. A total of 12 features were calculated from these measures.

- Spectral contrast

Spectral contrast is defined as the difference between valleys and peaks that compose the spectrum. The spectrogram is divided into sub-bands. For each sub-band, the energy contrast is estimated by comparing the mean energy in the top quantile (peak energy) to that of the bottom quantile (valley energy). High contrast values generally correspond to clear, narrow-band signals, while low contrast values correspond to broad-band noise.[25] Three features were derived from this measure: the mean, the maximum, and the standard deviation of the spectral contrast.

- Spectral roll-off frequency

The roll-off frequency characterizes the inclination of the signal's spectrum.[26] This feature is defined as the centre frequency for a spectrogram bin such that at least 85% of the energy of the spectrum is

contained in this bin and the bins below. Three features were computed using this measure: the mean, the maximum and the standard deviation of the spectral roll off frequencies.

- Spectral flatness

Spectral flatness quantifies how tone-like a signal is, as opposed to being a noise-like signal.[27] A high spectral flatness (closer to 1.0) indicates the spectrum is similar to white noise. Three features were calculated using this measure: the mean, the maximum and the standard deviation of the spectral flatness.

- Spectral centroid

The spectral centroid indicates the location of the centre of mass of each frequency bin in the spectrogram.[28] For each one of these measures three features were calculated: the mean, the maximum and the standard deviation.

References

1 Lyons, J. *et al.* Endogenous light scattering as an optical signature of circulating tumor cell clusters. *Biomed Opt Express* **7**, 1042-1050, doi:10.1364/BOE.7.001042 (2016).

2 Dasgupta, N. *et al.* Class-based target identification with multiaspect scattering data. *IEEE Journal of Oceanic Engineering* **28**, 271-282, doi:10.1109/joe.2003.811899 (2003).

3 Diggle, P. *Statistical analysis of spatial and spatio-temporal point patterns, third edition*. (2013).

4 Yang, D., Hong, K. S., Yoo, S. H. & Kim, C. S. Evaluation of Neural Degeneration Biomarkers in the Prefrontal Cortex for Early Identification of Patients With Mild Cognitive Impairment: An fNIRS Study. *Front Hum Neurosci* **13**, 317, doi:10.3389/fnhum.2019.00317 (2019).

5 Zhang, Y. *et al.* Integration of 24 Feature Types to Accurately Detect and Predict Seizures Using Scalp EEG Signals. *Sensors (Basel)* **18**, doi:10.3390/s18051372 (2018).

6 Pincus, S. M. Approximate entropy as a measure of system complexity. *Proc Natl Acad Sci U S A* **88**, 2297-2301, doi:10.1073/pnas.88.6.2297 (1991).

7 Molinari, F. *et al.* Entropy analysis of muscular near-infrared spectroscopy (NIRS) signals during exercise programme of type 2 diabetic patients: quantitative assessment of muscle metabolic pattern. *Comput Methods Programs Biomed* **112**, 518-528, doi:10.1016/j.cmpb.2013.08.018 (2013).

8 Hornero, R., Aboy, M., Abasolo, D., McNames, J. & Goldstein, B. Interpretation of approximate entropy: analysis of intracranial pressure approximate entropy during acute intracranial hypertension. *IEEE Trans Biomed Eng* **52**, 1671-1680, doi:10.1109/TBME.2005.855722 (2005).

9 Ocak, H. Automatic detection of epileptic seizures in EEG using discrete wavelet transform and approximate entropy. *Expert Systems with Applications* **36**, 2027-2036, doi:10.1016/j.eswa.2007.12.065 (2009).

10 Roberts, S. J., Penny, W. & Rezek, I. Temporal and spatial complexity measures for electroencephalogram based brain-computer interfacing. *Med Biol Eng Comput* **37**, 93-98, doi:10.1007/BF02513272 (1999).

11 Bachmann, M. *et al.* Methods for classifying depression in single channel EEG using linear and nonlinear signal analysis. *Comput Methods Programs Biomed* **155**, 11-17, doi:10.1016/j.cmpb.2017.11.023 (2018).

12 Al-Nuaimi, A. H., Jammeh, E., Sun, L. & Ifeachor, E. Higuchi fractal dimension of the electroencephalogram as a biomarker for early detection of Alzheimer's disease. *Annu Int Conf IEEE Eng Med Biol Soc* **2017**, 2320-2324, doi:10.1109/EMBC.2017.8037320 (2017).

13 Hosseinifard, B., Moradi, M. H. & Rostami, R. Classifying depression patients and normal subjects using machine learning techniques and nonlinear features from EEG signal. *Comput Methods Programs Biomed* **109**, 339-345, doi:10.1016/j.cmpb.2012.10.008 (2013).

14 Kesic, S. & Spasic, S. Z. Application of Higuchi's fractal dimension from basic to clinical neurophysiology: A review. *Comput Methods Programs Biomed* **133**, 55-70, doi:10.1016/j.cmpb.2016.05.014 (2016).

15 Khoa, T. Q., Ha, V. Q. & Toi, V. V. Higuchi fractal properties of onset epilepsy electroencephalogram. *Comput Math Methods Med* **2012**, 461426, doi:10.1155/2012/461426 (2012).

16 Gomez, C., Mediavilla, A., Hornero, R., Abasolo, D. & Fernandez, A. Use of the Higuchi's fractal dimension for the analysis of MEG recordings from Alzheimer's disease patients. *Med Eng Phys* **31**, 306-313, doi:10.1016/j.medengphy.2008.06.010 (2009).

17 Hurst, H. E. The Problem of Long-Term Storage in Reservoirs. *International Association of Scientific Hydrology. Bulletin* **1**, 13-27, doi:10.1080/02626665609493644 (1956).

18 Hjorth, B. EEG analysis based on time domain properties. *Electroencephalogr Clin Neurophysiol* **29**, 306-310, doi:10.1016/0013-4694(70)90143-4 (1970).

19 Cecchin, T. *et al.* Seizure lateralization in scalp EEG using Hjorth parameters. *Clin Neurophysiol* **121**, 290-300, doi:10.1016/j.clinph.2009.10.033 (2010).

20 Roberts, P. L. D., Jaffe, J. S. & Trivedi, M. M. Multiview, Broadband Acoustic Classification of Marine Fish: A Machine Learning Framework and Comparative Analysis. *IEEE Journal of Oceanic Engineering* **36**, 90-104, doi:10.1109/joe.2010.2101235 (2011).

21 Oweis, R. J. & Abdulhay, E. W. Seizure classification in EEG signals utilizing Hilbert-Huang transform. *Biomed Eng Online* **10**, 38, doi:10.1186/1475-925X-10-38 (2011).

22 Azimi-Sadjadi, M. R., Yao, D., Huang, Q. & Dobeck, G. J. Underwater target classification using wavelet packets and neural networks. *IEEE Trans Neural Netw* **11**, 784-794, doi:10.1109/72.846748 (2000).

23 Pereira, T., Paiva, J. S., Correia, C. & Cardoso, J. An automatic method for arterial pulse waveform recognition using KNN and SVM classifiers. *Med Biol Eng Comput* **54**, 1049-1059, doi:10.1007/s11517-015-1393-5 (2016).

24 Wang, X., Gong, G. & Li, N. Automated Recognition of Epileptic EEG States Using a Combination of Symlet Wavelet Processing, Gradient Boosting Machine, and Grid Search Optimizer. *Sensors (Basel)* **19**, doi:10.3390/s19020219 (2019).

25 Dan-Ning, J., Lie, L., Hong-Jiang, Z., Jian-Hua, T. & Lian-Hong, C. in *Proceedings. IEEE International Conference on Multimedia and Expo.* 113-116 vol.111.

26 Kos, M., Kačič, Z. & Vlaj, D. Acoustic classification and segmentation using modified spectral roll-off and variance-based features. *Digital Signal Processing* **23**, 659-674, doi:10.1016/j.dsp.2012.10.008 (2013).

27 Dubnov, S. Generalization of Spectral Flatness Measure for Non-Gaussian Linear Processes. *IEEE Signal Processing Letters* **11**, 698-701, doi:10.1109/lsp.2004.831663 (2004).

28 Klapuri, A. & Davy, M. *Signal Processing Methods for Music Transcription*. (2006).
